# Necrosis of the Jaw

**Published:** 1889-10-26

**Authors:** 


### NECROSIS OF THE JAW.

Mr. Clement Lucas, surgeon to Guy's Hospital, gives an account of two cases of necrosis of the alveolar processes following measles.‡ The one case was that of a child who had suffered from measles and inflammation of the lungs, and on admission to the hospital five weeks afterwards, the mouth and breath were found very fœtid. On the front of both the upper and lower maxillæ, and limited to the anterior or central part of both alveoli, there was a greenish-black portion of necrosed bone. Four incisor teeth had dropped out. In the lower jaw the necrosis, besides involving all the incisor sockets, included those of the two canines. The necrosed portion of the upper jaw was removed with forceps under chloroform with ease. The removal of the necrosed bone from the lower jaw was more difficult. The piece removed from the upper jaw was nearly quadrilateral, measuring threequarters of an inch both vertically and transversely, and it contained the sockets of the four incisor teeth. The portion removed from the lower jaw was larger, containing all the incisor sockets and the canine sockets. The operation was performed on July 2, and on the 7th the child was discharged convalescent.

The second case was that of a child, aged three, who had measles in August, 1882, and was ill for six weeks. The patient was admitted in February, 1883, and shortly before being brought to the hospital the alveolar process of the upper jaw corresponding to the four incisor teeth dropped out, while the alveolar process of the lower jaw became blackened and denuded of gum, while the breath was very offensive. The alveolar process of the lower jaw corresponding with the four incisor sockets and the right canine, and the alveolar process of the upper jaw bearing the sockets of the molar teeth on the left side were all removed, the patient rapidly recovering, and the gum granulating over.

These local affections of the jaws are supposed to be more frequently associated with debility from measles or scarlet fever than with weakness following any other variety of fever, but probably this is more apparent than real. For necrosis of the jaw is a malady which usually attacks children, and children are more frequently the victims of measles and of scarlet fever than adults. Whether or not there is any direct relation between these maladies and necrosis of the jaw, there is no doubt that both the former and the latter are seen most frequently in children. Mr. Lucas remarks on the similarity of the progress of the cases, both showing more rapid disease in the upper than in the lower jaw, and Mr. Lucas explains this by referring it to the heavier and denser texture of the lower maxilla. Necrosis may attack any part of the jaws, and in our experience the lower jaw is most frequently attacked. We are not aware that anyone has, as yet, investigated the after career of children who have suffered as detailed above after measles or scarlet fever. It would be well to ascertain what influence losses as suffered by Mr. Clement Lucas's patients, may have on future life.

